# The role of 3D printing in the fight against COVID-19 outbreak

**DOI:** 10.2217/3dp-2020-0028

**Published:** 2021-05-05

**Authors:** Payar Radfar, Sajad Razavi Bazaz, Fateme Mirakhorli, Majid Ebrahimi Warkiani

**Affiliations:** 1 ^1^School of Biomedical Engineering, University of Technology Sydney, Sydney, NSW, 2007, Australia; 2 ^2^Institute of Molecular Medicine, Sechenov University, Moscow, 119991, Russia

**Keywords:** 3D printing, additive manufacturing, COVID-19 outbreak, shortage of personal protective equipment

## Abstract

Along with the COVID-19 pandemic, urgent needs for medical and specialized products, especially personal protective equipment, has been overwhelming. The conventional production line of medical devices has been challenged by excessive global demand, and the need for an easy, low-cost and rapid fabrication method is felt more than ever. In a scramble to address this shortfall, manufacturers referred to additive manufacturing or 3D printing to fill the gap and increase the production line of medical devices. Various previously/conventionally fabricated designs have been modified and redesigned to suit the 3D printing requirement to fight against COVID-19. In this perspective, various designs accommodated for the current worldwide outbreak of COVID-19 are discussed and how 3D printing could help the global community against the current and future conditions has been explored.

The outbreak of the 2019 novel coronavirus disease (COVID-19) resulted in a viral pandemic affecting more than 200 countries around the globe [1–3]. COVID-19 is highly transmissible and primarily transmits through respiratory droplets, direct contact with an infected person, or contacting contaminated objects and surfaces [4,5]. The contagiousness and outbreak of COVID-19 has resulted in a significant shortage of personal protective equipment (PPE) and medical supplies. In March 2020, the WHO warned industries and governments to increase the manufacturing of PPE and medical supplies by 40% to meet the rising global demand; caused by rising need, panic buying, hoarding and misusing, which is putting lives at risk globally [6,7]. The rational use of PPE in healthcare settings, including gloves, goggles, face shield and gowns, is crucial to allow treatment of patients without further transmission of the disease [8–10]. Considering the instant and high global manufacturing demand of PPE and medical supplies, additive manufacturing, also known as 3D printing [11,12], is a perfect candidate for rapid prototyping and production of them [13–16]. Herein, the critical role of additive manufacturing during the COVID-19 pandemic is discussed, and is exploited how 3D printing can aid the global community in future critical events that highly disrupt supply chains. Further, several key designs are reported that were used to fight the outbreak of coronavirus, followed by a discussion about the future direction of how 3D printing and rapid prototyping can benefit people around the globe to control future pandemics. The 3D printed devices were categorized based on their functionality into prevention, treatment and diagnosis devices. Moreover, in the Supplementary Information, the most updated computer-aided designs of the proposed devices have been provided.

## Role of 3D printing in pandemics

**Figure 1.**
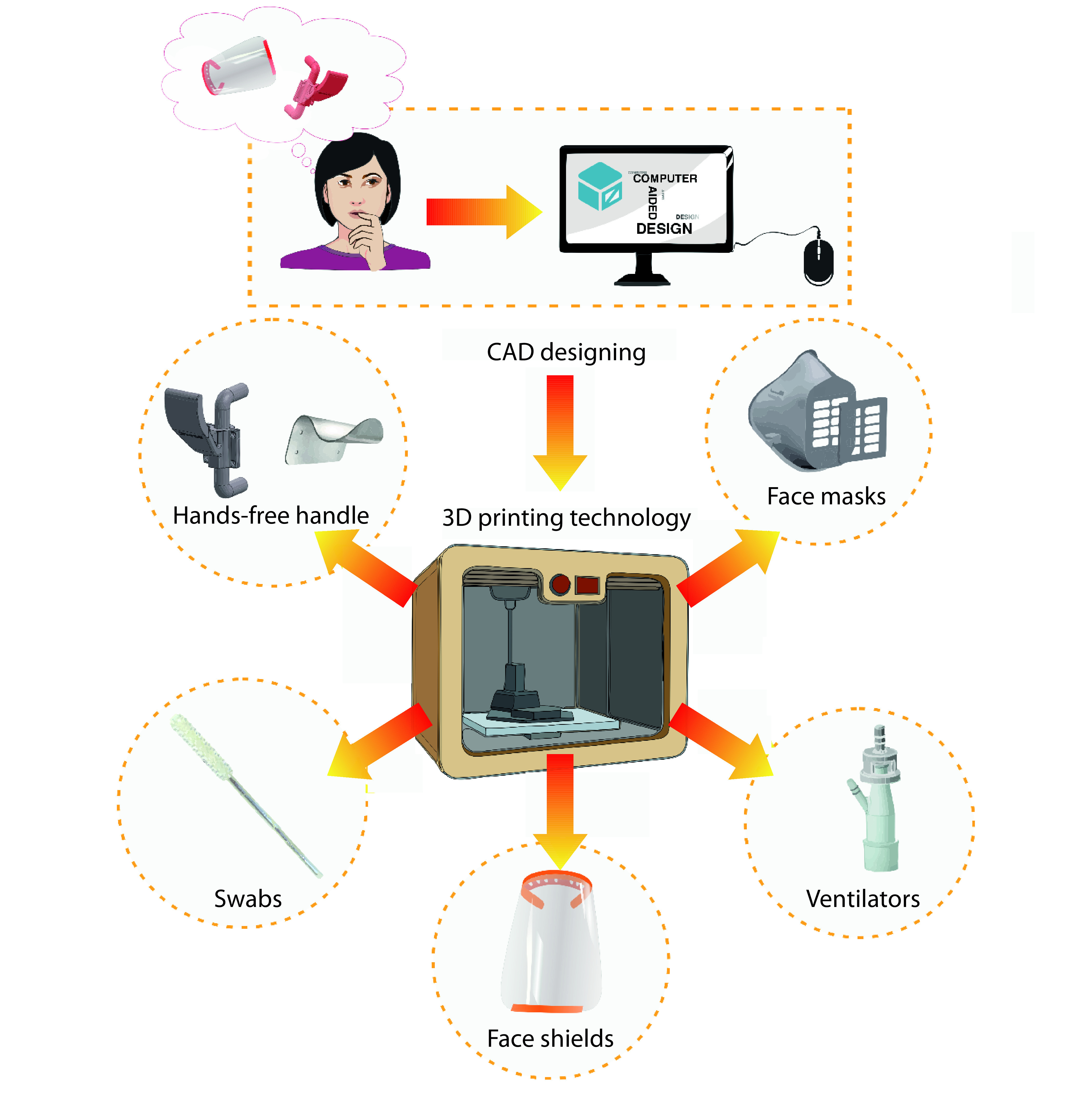
Illustration of the 3D printing process from brainstorming to computer-aided design modeling and fabrication of various parts that have been created to fight against the COVID-19 pandemic. The typical devices developed and used in the early days of the pandemic include: hands-free handle, face masks, ventilators, face shields and swabs. CAD: Computer-aided design.

The accessibility and fast prototyping capabilities of 3D printing have crucially helped the global shortages of medical and protective equipment. People with access to 3D printers assisted the fight against the COVID-19 pandemic by designing and producing preventive, diagnosis and treatment devices from brainstorming to implementation instantly (Figure 1) [17]. For this purpose, people are gathering globally in online communities to share/exchange ideas and designs.

Generally, 3D printing works based on creating objects by stacking layers of materials to build a desired part. Compared with subtractive manufacturing processes, which generally waste 80–90% of the raw material, 3D printing offers less material wastage (depending on the 3D printing method, material wastage includes support materials, base layers or exposed powder beds) [13]. Over the past decades, 3D printing technologies have substantially aided designers to rapidly meet their client’s demands by reducing the manufacturing lead time and material wastage [18–20]. 3D printing can mainly be classified into (but not limited to) the following groups: two-photon polymerization, stereolithography, fused deposition modeling, digital light processing, selective laser sintering and MultiJet printing. Based on working principles, each 3D printing approach has its own advantages and disadvantages, which are compared and discussed in detail in Table 1. Generally, stereolithography has the highest resolution, fused deposition modeling is the most common inexpensive method, and MultiJet printing is well-known for its biocompatibility, biodegradability and mechanical properties [21–23]. Apart from the printing method, 3D printers brought a paradigm in rapid prototyping and allow accessible manufacturing to everyone at a reasonably low setup and printing cost, especially for globally critical situations, similar to this pandemic, when time and minimum human interaction (social distancing practices) play important roles [24–26]. Table 2 provides a qualitative comparison between different fabrication methods showing that injection molding is not a suitable approach for rapid prototyping and low volume fabrication. However, large-scale production of parts through injection molding is the preferable approach when the lead time is not the primary concern [27], unlike the critical global events that design to fabrication time is of vital importance. Furthermore, computerized numerical control (CNC) machining is a widely accepted manufacturing method that is well known for wide material selection, reasonable lead time. Also, CNC machining is suitable for low-to-mid-volume fabricating with high precision; however, the high machinery costs and requirement for trained operators limits the applicability of this method during critical events [28,29]. It is also worth mentioning that although 3D printing has significantly helped in rapid manufacturing of PPE during the pandemic, the environmental impact of material wastage of 3D printing should be considered, especially in large-scale global production.

**Table 1.** Comparison of fabrication methods based on working principles, resolution quality, advantages and disadvantages.

Printing process	Method	Material	Resolution	Pros	Cons
SLA	Vat polymerization by UV laser beam	Photo-curable resin polymer	Layers: 0.01–0.50 mm Features: 0.1 mm Surface: smooth Speed: average	Fine details Easy post processing Smooth surface finish Low material consumption Barely visible	Relatively weak Susceptible to lights and heat Longer than DLP Limited life span
DLP	Vat polymerization through UV light	Photo-curable resin polymer	Layers: 0.01–0.50 mm Features: 0.1 mm Surface: smooth Speed: average	Fine details Easy post processing Smooth surface finish Less running cost than SLA	Relatively weak Susceptible to lights and heat Limited life span
SLS	Powder bed fusion	Metals, alloys and certain polymers powder	Layers: 0.06–0.15 mm Features: 0.3 mm Surface: rough Speed: fast	Strong Large build plate Excellent layer adhesion Ideal for dyeing	Prone to shrinkage Difficult post processing Excessive wastage Relatively expensive
FDM	Material extrusion	Filaments	Layers: 0.1–0.3 mm Surface: very rough Speed: slow	High part strength Low cost Broad range in material Lower post processing time Accessibility Easy to use	Poor surface finish Slow printing process Prone to warping and shrinkage
2PP	2PPs	Photo-curable resin polymer	Layer: down to nm Surface: very smooth Speed: very low	Excellent for complex designs Excellent surface smoothness	High cost Not suitable for more than ∼3-mm-height designs
MultiJet	Material jetting	Precision plastics, elastomeric, multi-material composites and wax	Layers: 0.01–0.50 mm Features: 0.02 mm Surface: smooth Speed: fast	Full color Materials with different young modulus	Poor mechanical properties Very expensive
Injection molding	Injection of molten materials	Nylon, polycarbonate and polystyrene	Excellent surface finishing Tolerance: 0.040–0.060 mm	Suitable for high volume production Wide range of material selection	High cost for low volume production Excessive long lead time
CNC machining	Material removal	Aluminium, brass and magnesium	Smooth surface finishing Tolerance: 0.020–0.040 mm	Broad material selection Acceptable turnaround time	Not suitable for complex designs High equipment cost

2PP: Two-photon polymerization; CNC: Computerized numerical control; DLP: Digital light processing; FDM: Fused deposition modeling; SLA: Stereolithography; SLS: Selective laser sintering.

**Table 2.** Qualitative comparison of different fabrication methods based on, cost for low & high volume, lead time, material selection, surface finish and complexity.

		SLA^†^	DLP^‡^	SLS^§^	FDM^¶^	MultiJet	2PP^#^	Injection molding	CNC machining^††^
Cost for low volume									
Cost for high volume									
Lead time									
Material selection									
Surface finish									
Design complexity									
Scale bar:									
(1)	(2)	(3)	(4)	(5)	(6)	(7)	(8)	(9)	(10)

† SLA.

‡ DLP.

§ SLS.

¶ FDM.

# 2-PP.

†† CNC machining.

2PP: Two-photon polymerization; CNC: Computerized numerical control; DLP: Digital light processing; FDM: Fused deposition modeling; SLA: Stereolithography; SLS: Selective laser sintering.

## Protection

With the outbreak of novel COVID-19 in late 2019, most countries have been facing serious difficulties with the supply of protective devices among general public and healthcare workers [30,31]. The implementation of infection preventing methods is of great importance in healthcare settings in order to control further spread of this disease [26,32,33]. Furthermore, for controlling the spread of COVID-19, the protection is not only limited to the healthcare workers but is also essential for any person working in public, especially those who directly deal with and provide in-person services [34,35]. Given the shortage and importance of protective equipment, designers from all over the world have proposed different 3D-printed devices to help prevent the spread of this virus and address global needs [36]. As a part of the global response, face shields were one of the most popular prevention solutions among designers that provide a physical barrier between the user’s face and surroundings. Face shields are one of the airborne precaution pieces of equipment that the WHO has recommended as part of PPE to avoid transmission of COVID-19 and other similar infectious diseases [8,37]. Multiple 3D-printing companies, including Formlabs, Prusa and Stratasys, are providing design protocols for manufacturing of face shields and sharing their designs online [38–41]. The majority of the current face shield designs consist of a 3D-printed headband that fits the user’s head, and it is designed to hold a visor/front plate that can be made of any laser cuttable clear plastic [40]. Although face shields are reusable and durable, there are some serious concerns regarding their disinfection after every usage, and there are clear protocols provided to disinfect them [42]. Also, face shields do not eliminate the protection need of eyes (i.e., goggles), and the WHO still recommends the use of goggles and face shields simultaneously [8]. Hence, some designers have proposed larger face shields that allow enough space for wearing personal goggles [43,44].

Also, with the global shortage of masks, designers have focused on alternative ways to meet this demand. A great effort has been allocated to repurpose scuba and snorkel masks to face masks using a conversion [45–47]. This conversion can hold and connect a filter to the mask, which is small in size and can be 3D-printed rapidly at a low price. However, it does require the user to have access to the mask and filter papers to fit inside the holder. Also, similar to face shields, this mask is reusable, and there is a need for disinfection of them after every usage. Retrofitting everyday equipment such as scuba diving masks to PPE is one of the ways to battle the COVID-19 pandemic and such creativities of designers can benefit global society.

**Figure 2.**
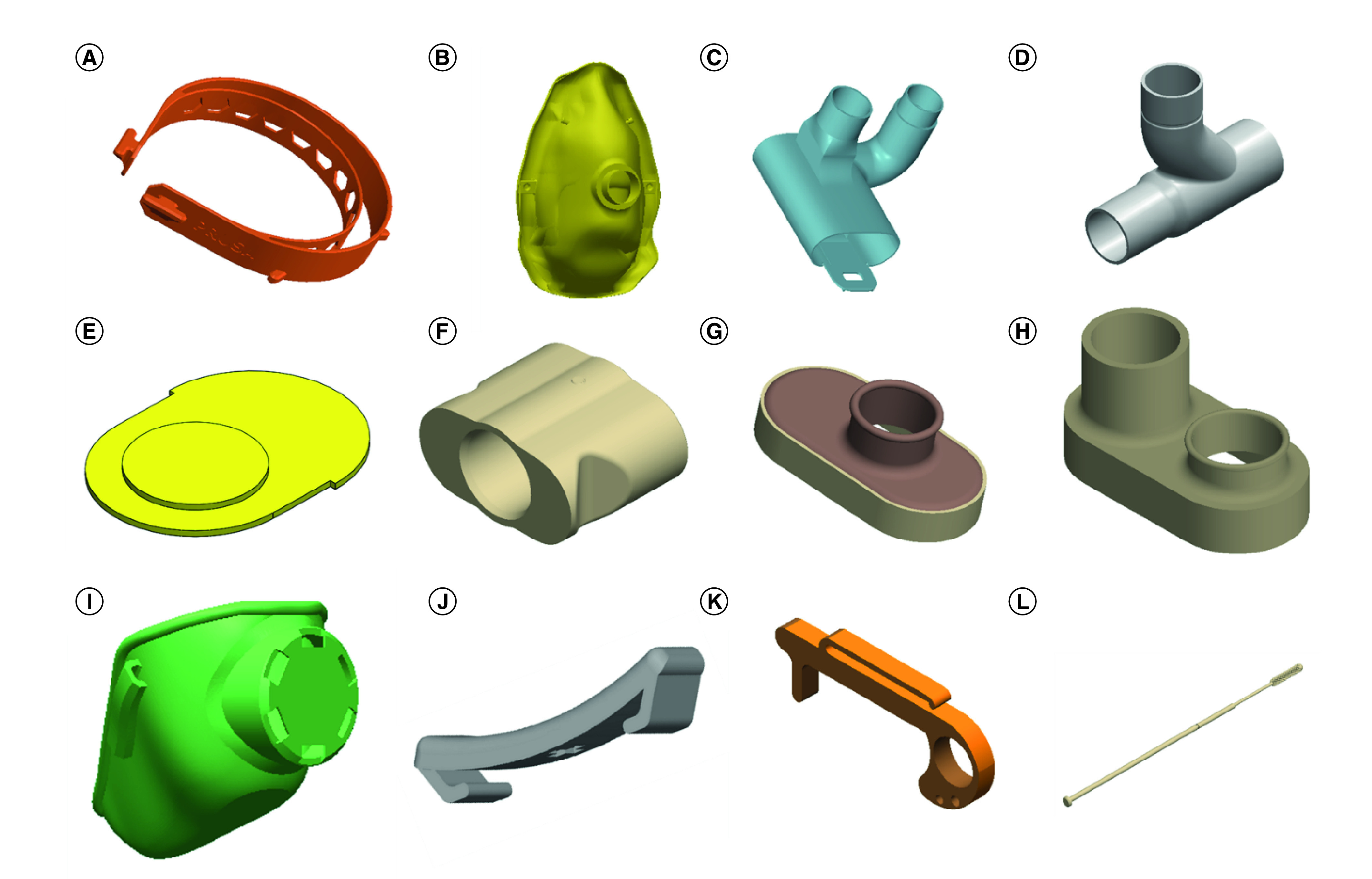
Devices designed and developed for COVID-19 pandemic situation. 3D-printed parts to help fight against COVID-19 including: **(A)** face shield (the holder/frame only is 3D printed) **(B)** mask **(C)** scuba diving conversion adaptor to ventilator **(D)** scuba diving adaptor **(E)** face shields **(F)** scuba conversion to N95 **(G)** valve **(H)** body valve **(I)** whole mask **(J)** mask holding clip **(K)** doorhandle **(L)** swab.

Protective devices are not limited to masks or other physical barriers. Avoiding contact with potentially contaminated surfaces can significantly slow the spread of COVID-19 [35,48,49]. Therefore, different gadgets/tools have been developed to minimize direct contact of surfaces. The hands-free door handle attachment is one of the gadgets that can allow opening doors using elbows/feet and can simply be 3D printed to reduce direct contact with the door handle as a potential point of contamination [36]. Also, small gadgets can be 3D printed to be used for pressing buttons in public areas or holding small objects, as shown in Figure 2. Additionally, there are other designs focused on enhancing the ergonomics for healthcare staff and other continuous mask users with a simple clip which holds the mask bands at the back part of the head and reduces the stress on ears.

## Treatment/hospital care

With the current outbreak of COVID-19, there is a serious demand for intensive care at hospitals, and multiple countries have experienced shortage of key hospital equipment, including ventilators [50,51]. The 3D-printed devices are utilized to aid the treatment of patients in hospitals. Based on our extensive research, the majority of 3D-printed inventions/designs lie around providing respiratory care for patients. Similar to protection devices, scuba diving masks were shown to be converted to ventilators using a conversion that can be connected to oxygen tanks [39,52].

Additionally, earlier in 2008, G Neyman and Charlene Babcock designed and reported a simple splitting device for the parallel use of single ventilators for multiple patients in emergency situations [53]. Inspired by that idea, Formlabs, along with other companies, designed and produced tubing splitters, suitable for the parallel use of a single oxygen mask [39,54].

## Diagnosis

Diagnosis and isolation of COVID-19 patients are among the main ways to fight the current pandemic [55,56]. Most of the SARS-CoV-2 testing methods use a nasal or mouth swab to collect clinical test samples of nasal/mouth secretions from the back of the nose and throat, when then the collected sample can be tested. During this pandemic, nasal swabs were used drastically to meet the need for large-scale virological testing which resulted in an acute shortage of them across the globe, especially in the early days of this disease [57–59]. Different companies and organizations, including Formlabs, Harvard University, Massachusetts Institute of Technology, etc., have designed and fabricated nasal swabs using 3D printed material to overcome this shortage [60]. These swabs are designed to collect saliva or nasal secretions’ samples from the mouth and nose. Collected samples could then be used for testing of COVID-19 using different approaches such as nucleic acid amplification or immunoassay detection approaches [61,62]. All the information regarding the swab’s design and operating instructions are available on the GitHub platform with a single and common goal: ‘stopping this pandemic’ [63]. While the nasal swabs are a relatively simple device, they need to be thin, long and flexible enough to reach deep into the nasal cavity in order to avoid producing false-negative results [60,64,65]. Formlabs worked closely with USF health and Northwell Health labs to test the prototypes of their nasal swabs. They successfully completed a clinical validation and will continue producing swabs in its US FDA-registered, ISO13485 certified facility in the USA (more information is provided on their website) [66]. Figure 2 shows different 3D-printed parts for fighting COVID-19 pandemic, which were discussed throughout this review.

## Conclusion

The outbreak of COVID-19 hugely impacted our lifestyles during the past couple of months, and the shortage of medical and preventive supplies was one of the main concerns globally to control and avoid the further spread of this virus. While most governments and companies were unable to provide critical supplies during this pandemic, designers around the world used online communities to provide potential designs and address people’s needs. Owing to the recent advancements of additive manufacturing, 3D printers were extensively used to build the proposed designs as emergency prevention, diagnosis and treatment devices in a rapid manner. Collaboration of big universities and key 3D printer making companies allowed for prototyping and quality testing new devices shared online for public use. The 3D printed nasal swabs were one of the main outcomes of such collaborations during the early stages of this pandemic [67,68]. Despite the rapidness and accessibility of 3D printing, one of the main challenges with the mentioned approach is the lack of safety regulation and meeting the standards of proposed devices. Further involvement of governments and authorities can provide a standard process for regulatory checking of the designs and clinical applications of 3D printed medical devices.

Although these efforts did not meet the entire demands, both in quantity and quality, it was a significant step in showcasing the power and potentials of rapid prototyping and additive manufacturing. With the advancement of technology and expansion of engineering discipline use in medical industries, it is believed that in future crucial improvements can be observed in patient care settings. The unfortunate spread of coronavirus and the quick response of designers around the globe revealed the importance of 3D printing in rapid and low-cost manufacturing of critical tools. In the upcoming years, there is potential to see a greater support from governments and global society for the development of manufacturing approaches capable of meeting user demands at a low cost and little turnaround time.

Lastly, point-of-care devices have been one of the major needs during the recent COVID-19 pandemic and the dramatic impact of early detection of infectious diseases in controlling outbreaks has been reported [69]. Considering the importance of early detection and isolation of infected people, a wide range of researchers and companies across the globe have been proposing novel detection devices/protocols based on different principles, including immunoassay and molecular based approaches [70,71]. In the past, multiple researchers have shown the possibility of implementing 3D-printing technologies as a tool to make point-of-care platforms that could sync and work with smartphones to allow detection of certain diseases at the point of need with minimal training and required equipment [72,73]. These all-in-one point-of-care devices allow a user-friendly detection that would be suitable for developing countries due to the low cost and accessibility of 3D printing. Given the necessity of detecting infected people during a pandemic, 3D printing can play a crucial role in the fabrication of point-of-care devices for current and future pandemics.

## Future perspective

The occurrence of the COVID-19 pandemic alerted the world to how sudden product demand can challenge the supply chain to an extent where even first-world countries faced serious difficulties in addressing the basic hygienic needs of people. Besides the excessive need for items including PPE and groceries, the pandemic’s mental pressure on the general public caused significant panic buying and hoarding of different products. Additionally, despite the advancements in the world’s healthcare ecosystems, the COVID-19 pandemic brought a clear message concerning the vulnerability of the global community to such global crisis, with subsequent economic crisis. Since re-occurrence of these critical situations might be expected in the future, accessibility to in-house manufacturing techniques, including 3D printing, can provide people with alternative solutions, helping to control the impact of a sudden increase in product demand.

However, current 3D printing technologies face several critical shortcomings that prevent their everyday usage. First, the current technologies require engineering skills and expertise to design and model the computer-aided design files. Second, most of the 3D printed parts, regardless of the printing method, require post processing such as support removal, washing and postcuring, which all require prior knowledge. Third, 3D printers require on-going maintenance including cleaning, calibration and replacement of consumables as well as disposable accessories which all contribute to hidden manufacturing costs. Finally, large-scale production of parts with the current 3D printing speed and setting is constrained by cost and production time which is the most challenging issue during the pandemic situation. Therefore, large-scale costs and production time needs to significantly improve before 3D printers can be implemented as a mass manufacturing solution.

Biocompatibility of materials is an important aspect for fabrication and use of PPE. Although in-house 3D printing of PPE can provide an alternative manufacturing approach during high-demand periods, decentralized fabrication can lead to unregulated use of the tools and hence might raise safety concerns about the wrong material selection. To address this, regulatory agencies should provide a controlled database of approved designs and accurate safety information/protocols for public users. This also eliminates the potential issues involved with infringement of intellectual properties of manufacturing companies.

**Executive summary**

**Introduction**

- The COVID-19 outbreak resulted in a global pandemic and affected over 200 countries.
- The pandemic resulted in a serious shortage of personal protective equipment (PPE) and medical supplies.
- The WHO warned industries and governments to increase the manufacturing of PPE and medical supplies by 40% to meet the rising global demand; caused by rising need, panic buying, hoarding and misusing, which is putting lives at risk globally.
- 3D printing and rapid prototyping played a critical role in aiding the fight against COVID-19.

**Role of 3D printing in pandemics**

- Accessibility and fast prototyping capabilities of 3D printing allowed for in-house fabrication of high-demand PPE and medical devices.
- People from across the globe shared and exchanged design ideas and protocols for creating protective, hospital care and diagnostic devices.
- Different 3D printing technologies and fabrication have been introduced and compared in the context of the fight against pandemic.

**Protection**

- Key designs that were developed for reducing direct interaction of people were discussed.

**Treatment/hospital care**

- Key devices developed for aiding the hospitalization of patients, especially in the settings where ventilators were critically needed.

**Diagnosis**

- In the early days of the pandemic, the great need for medical swabs for sample collection led to a shortage of these products.
- The 3D printed swabs developed by different companies and institutes were tested and used to overcome this shortage.

**Future perspective**

- The lessons learnt from this pandemic have been discussed.
- The role of 3D printing in such critical situations has been evaluated.
- The shortcomings of 3D printing technologies have been discussed in terms of large-scale production and its corresponding cost and time.
- The regulatory aspects of 3D printed medical devices and PPEs have been discussed including; material biocompatibility, safety issues and IP infringement.
